# Clinic Attendance for Antiretroviral Pills Pick-Up among HIV-Positive People in Nepal: Roles of Perceived Family Support and Associated Factors

**DOI:** 10.1371/journal.pone.0159382

**Published:** 2016-07-20

**Authors:** Rakesh Ayer, Kimiyo Kikuchi, Mamata Ghimire, Akira Shibanuma, Madhab Raj Pant, Krishna C. Poudel, Masamine Jimba

**Affiliations:** 1 Department of Community and Global Health, Graduate School of Medicine, The University of Tokyo, 7-3-1 Hongo, Bunkyo-ku, Tokyo, 113–0033, Japan; 2 Department of Health Care Policy and Management, Graduate School of Comprehensive Human Sciences, University of Tsukuba, 1-1-1, Tennoudai, Tsukuba, Ibaraki, 301–8577, Japan; 3 Antiretroviral Therapy Clinic, Tribhuvan University Teaching Hospital, Maharajgunj, Kathmandu, Nepal; 4 Department of Health Promotion and Policy, School of Public Health and Health Sciences, University of Massachusetts Amherst, 316 Arnold House, 715 North Pleasant St, Amherst, MA, 01003–9304, United States of America; Tulane University School of Public Health, UNITED STATES

## Abstract

**Introduction:**

HIV-positive people’s clinic attendance for medication pick-up is critical for successful HIV treatment. However, limited evidence exists on it especially in low-income settings such as Nepal. Moreover, the role of family support in clinic attendance remains under-explored. Therefore, this study was conducted to examine the association between perceived family support and regular clinic attendance and to assess factors associated with regular clinic attendance for antiretroviral pills pick-up among HIV-positive individuals in Nepal.

**Methods:**

A cross-sectional study was conducted among 423 HIV-positive people in three districts of Nepal. Clinic attendance was assessed retrospectively for the period of 12 months. To assess the factors associated, an interview survey was conducted using a semi-structured questionnaire from July to August, 2015. Multiple logistic regression models were used to assess the factors associated with regular clinic attendance.

**Results:**

Of 423 HIV-positive people, only 32.6% attended the clinics regularly. They were more likely to attend them regularly when they received high family support (AOR = 3.98, 95% CI = 2.29, 6.92), participated in support programs (AOR = 1.68, 95% CI = 1.00, 2.82), and had knowledge on the benefits of antiretroviral therapy (AOR = 2.62, 95% CI = 1.15, 5.99). In contrast, they were less likely to attend them regularly when they commuted more than 60 minutes to the clinics (AOR = 0.53, 95% CI = 0.30, 0.93), when they self-rated their health status as being very good (AOR = 0.13, 95% CI = 0.04, 0.44), good (AOR = 0.14, 95% CI = 0.04, 0.46), and fair (AOR = 0.21, 95% CI = 0.06, 0.70).

**Conclusion:**

HIV-positive individuals are more likely to attend the clinics regularly when they receive high family support, know the benefits of antiretroviral therapy, and participate in support programs. To improve clinic attendance, family support should be incorporated with HIV care programs in resource limited settings. Service providers should also consider educating them about the benefits of antiretroviral therapy.

## Introduction

Antiretroviral therapy (ART) has changed the landscape of the HIV epidemic. In 2000, access to ART was limited to only 2% of the people living with HIV in the world [1]. However, in the last decade, ART had been rapidly scaled up [1–3] and led to more than 15 million (40%) HIV-positive individuals to receive ART [1]. To sustain these achievements, the World Health Organization (WHO) has recommended a treat-all strategy, in which all HIV-positive individuals should receive ART immediately after HIV diagnosis [4]. Consequently, ART has converted HIV infection into a chronic manageable infection [5, 6].

To suppress HIV infection, people living with HIV should be involved with HIV care continuously [7]. It includes regular attendance to ART clinics for multiple purposes, such as antiretroviral (ARV) pills pick-up, treatment of opportunistic infections, CD4 cell count, and viral load tests. Above all, pills pick-up is the major purpose for attending clinics. Inconsistent clinic attendance for pills pick-up may directly impair ART adherence and worse treatment outcomes such as virologic failure [8]. Their high mortality rates have been also associated with inconsistent clinic attendances [9, 10].

Several factors are known to influence HIV-positive individuals’ clinic attendance for pills pick-up. Among them are unemployment, low literacy, younger age, and HIV status disclosure [11]. Moreover, a variety of factors have been identified, such as transportation costs, long commuting time to clinics [12], ART literacy [13, 14], presence of opportunistic infections, such as Tuberculosis-HIV co-infection, their health status [11], side-effects and drug toxicities of ART regimens [15], and their involvement to support programs [16].

In addition to these factors, family support has a potential of low-cost, high-impact on ensuring regular clinic attendance. As ART involves life-long medication procedure, HIV-positive individuals’ continuous adherence to ART depends on their social and family support [17–19]. It may play vital role in treatment success, reduction in ARV drug resistance, and HIV- positive individuals’ improved quality of life [17, 20].

In 2015, the Asia and Pacific region hosted 5.1 million HIV-positive people. The HIV epidemic in this region is concentrated among key populations such as people who inject drugs and men who have sex with men. In the context of HIV treatment, this region has made significant progress by increasing ART coverage. The coverage was more than doubled in the last five years from 19% in 2010 to 41% in 2015 [21].

Similar to other Asian countries, in Nepal, the HIV epidemic is concentrated among high risk groups, such as seasonal migrants, people who inject drugs, and female sex workers. In 2015, HIV prevalence was 0.2% among adults and total 40,000 HIV-positive individuals were estimated to live [22]. In total, about 10,000 people living with HIV were accessing ART from 61 ART clinics throughout the country [23, 24]. Although most of ART services are provided free of cost, geographical difficulties, transportation, and opportunity costs are unexpectedly high which may jeopardize regular attendance to ART clinics [25, 26].

Regular clinic attendance, however, is necessary to ensure the success of Nepal’s ART program goals. According to the national ART guidelines 2012 and 2014 [27, 28], the Government of Nepal needs to achieve four major goals on ART program:

1. maximal and durable suppression of viral load;
2. restoration and/or preservation of immunologic function;
3. reduction of HIV morbidity and mortality, and
4. to improve quality of life of people living with HIV.

To achieve these goals, regular clinic attendance for pills pick-up is indeed a key to success.

To improve regular clinic access, several barriers must be identified and overcome. In the context of HIV care in Nepal, factors such as ART adherence, survival, and nutritional status had been explored [25, 26, 29–32]. No evidence was available on regular clinic attendance for pills pick-up. Additionally, the association of perceived family support with regular clinic attendance for pills pick-up remains unknown. This is partly because little attention has been given to the influence of family support upon clinic attendance for collecting medication. In this study, we assessed the magnitude of HIV-positive individuals’ regular clinic attendance for ARV pills pick-up and its association with perceived family support. We also assessed, factors associated with regular clinic attendance for antiretroviral pills pick-up among HIV-positive individuals in Nepal.

## Methods

### Study design

A cross-sectional study was conducted among HIV-positive individuals attending ART clinics in July and August, 2015. To assess the associated factors for regular clinic attendance, face to face interviews were conducted using a semi-structured questionnaire. Furthermore, retrospective assessment was performed to calculate their regular clinic attendance for mediation collection during the past twelve months. For that, data were collected on clinic attendance for pills pick-up from the treatment and care files of HIV-positive individuals.

### Study area

This study was conducted at three public ART clinics in the Chitawan, Kaski and Rupandehi districts of Nepal. During data collection period, 61 ART clinics were under the control of National Center for AIDS and STD Control / Ministry of Health and Population in five development regions. These study sites were selected based on following two characteristics of these ART clinics. Firstly, they had similar socio-economic background, and secondly, they dealt with higher number of people living with HIV accessing ART from these three clinics when compared with other clinics.

Selected clinics were located in the central and western development regions. According to the national census in 2011, approximately 600,000, 500,000, and 900,000 people live in Chitawan, Kaski, and Rupandehi districts, respectively [33]. One clinic was located in each of the three study districts. The first clinic was located in Bharatpur hospital of Chitawan district, the second one in the western regional hospital of Kaski district, and the final one in Lumbini zonal hospital of the Rupandehi district. According to the ART clinics about 2,000 HIV-positive individuals (20% of the whole country) [24] were under the ART program at these three clinics during the study period.

This study was undertaken in the aftermath of the earthquakes in Nepal that occurred in April and May, 2015. However, the study districts were among the least affected areas. During the study period, all hospitals and ART clinics under this study were functioning at their full capacity, suggesting those disasters had minimal impact on participants’ clinic attendance. This conclusion was also reached after seeking information from each HIV-positive individual about any injury or damage to themselves, to their family member’s life, and as well as to their property. Furthermore, each HIV-positive individual was asked about any hindrance they faced in their mobility or interruption to ART services after earthquakes. In the targeted districts, none of the people living with HIV reported any injury, loss of property, or any barrier in seeking ART services due to the earthquake.

### Participants’ inclusion criteria

Participants of this study were defined as people living with HIV aged more than or equals to 18 years old. They were also registered and accessing ART services from the three clinics for at least 12 months. This study included only those who were on ART according to the information obtained from the clinics by the end of June, 2015. Also, this study included only those who were receiving the first line treatment (because of very low number of HIV-positive individuals on the second line treatment). Participants were limited to those who started taking ART between July 2012 and June 2014. Their clinic attendance for pills pick-up was assessed during a period of 12 months, starting from July, 2014 to June, 2015. In ART clinics, HIV-positive individuals usually have to come for pills pick-up once a month. In some circumstances, their friends or family members’ pick-up pills on their behalf. During clinic visit, their bodyweight and medication adherence level are also recorded.

According to the medical records at the clinics, 1,048 people living with HIV started taking ARV during this period (July 2012 to June 2014). Out of which, 110 were less than 18 years old and were excluded from study. From the remaining 938, 353 were also excluded from analysis: 113 died, 81 were lost to follow up, and 159 were transferred to other ART clinics. In total, at the end of June 2015, 585 HIV-positive individuals were eligible. From the 585, 450 (77%) were recruited within the study period.

### Measurements

#### Clinic attendance for ARV pills pick-up

Clinic attendance adherence rate was assessed based on the rate of total days adhered among total scheduled days [34]. An attendance was called ‘adhered’, when they maintained attendance in less than or equals to 2 days among scheduled days [28]. Additionally, an attendance was also called ‘adhered’ when they maintained it up to 3 days earlier to the scheduled day. Information on clinic attendance was retrieved from HIV-positive individuals’ treatment and care files.

On time pills pick-up target was more than or equals to 90% according to the WHO’s guideline. This percentage is considered as appropriate level for viral suppression and to prevent HIV drug resistance [35]. National consolidated guidelines for treating and preventing HIV in Nepal also recommends that all pills should be collected with no more than a 2 day delay [28]. Dichotomous measure was used to assess the clinic attendance for pills pick-up. For this study, a 90% -100% [11, 36–39] range was used to define regular clinic attendance. In a similar setting, a study demonstrated strong association with HIV-positive individuals’ keeping more than 90% clinic attendance and their 100% treatment adherence [38]. To be consistent with a study in similar setting [38], they were categorized into regular and inconsistent attendants based on their twelve months’ clinic attendance adherence rate. Their keeping more than 90% (less than, or equals to 1 missed day) of their scheduled attendances were categorized as regular attendance and those with less than or equals to 90% (more than 1 missed days) attendance as inconsistent attendance [36, 38].

Clinic attendance for pills pick-up was usually scheduled for once in a month. In special circumstances, it could also be scheduled once every two months, or more, as well. HIV-positive individuals were clearly informed about the date of their next visit. The date of next visit was scheduled in a manner that it did not coincide with national holidays or weekends. Furthermore, in clinics there was no provision of cancelling a scheduled visit for pills pick-up. Information related to such cancel visit was not collected. This study assessed attendances only for pills pick-up and did not collect information on attendance when they were sick or any other visits.

#### Socio-demographic variables

Variables such as age, sex, formal education, and employment status were included in the questionnaire. These variables were adopted from the national demographic and health survey [40]. HIV-positive individuals’ place of residence was categorized into “rural” and “urban”. The place of residence was called “rural” when they reported living in village development committee. Their place of residence was called “urban” when they reported living in municipalities. This was adopted from the definition of national population and housing census 2011, Central Bureau of Statistics, Nepal [33]. Information was also collected on commuting time from their place of residence to the clinics. Furthermore, commuting time was categorized into ≤ 60 minutes and >60 minutes [25].

#### Health, HIV status and ART literacy related variables

A self-assessed health status was used to assess the general health status of HIV-positive individuals. This is a widely used global measure to assess health status of people. A single-item measure was administered to all people living with HIV. They were asked to answer the question, *“How is your health in general*?*”* rating their status as ‘excellent’, ‘very good’, ‘good’, ‘fair’, or ‘poor’. Studies had shown this measure as predictive of major health events and related behavior [41, 42].

This study also assessed the HIV status disclosure of HIV-positive individuals. A single question measure was used to assess this, *“Have you disclosed your HIV-positive status to anyone other than health care providers*?*”*. Their response was categorized into “Yes”, when they reported disclosure and “No”, when they did not report [25].

HIV-positive individuals’ medical records were used to collect information related to their HIV status. Information collected was related to clinical stage and ART regimens. WHO clinical staging criterion was used to collect information on clinical staging. This criterion is used to determine progression levels of HIV infection (from stage I to IV) [27]. Information related to TB diagnosis was also collected from HIV-positive individuals.

Furthermore, this study assessed HIV-positive individuals’ participation in supporting programs such as community and home based care program [43]. Additionally, ART literacy was also assessed using three questions developed from ART literacy course handbook for community-based caregivers [44].

#### Perceived family support

To measure perceived family support, we used the 10-item Nepali Family Support and Difficulty Scale developed by Kohrt, 2009 [45]. This scale was designed especially for the use in Nepal. It has been used widely in different peer reviewed literatures [19,46,47]. For each item, participants were asked to rate how true each statement is for their own family on a four-point Likert scale ranging from “Not at all” (0) to “All the time” (3). All the items were summed up to calculate total scores. Higher scores indicated higher perceived family support. Total score ranged from (0–30). Further, scores were categorized into low (0–22), moderate (23–26), and high (27–30) levels of perceived family support [47]. In this study, Cronbach’s alpha of this scale was 0.76.

### Data collection

Ten field researchers were recruited for data collection. Four field researchers were professional health assistants and six were experienced working in HIV-related programs. Training was conducted to clarify field researchers about study objectives, research ethics, and contents of questionnaire.

Data collection was done using face to face interviews between July and August, 2015. This study recruited 450 HIV-positive individuals during the study period, of them 423 were included in the final analysis; 27 were excluded due to incomplete and missing data. Furthermore, information related to clinic attendance and HIV status was retrieved from their treatment and care files. ART number of each HIV-positive individual was used to retrieve such information. The principal researcher checked the data consistency and completeness.

### Questionnaire

Semi-structured questionnaire (S1 Appendix) was prepared for data collection based on previous studies[11, 12, 17, 26, 36]. Questionnaire was first prepared in English and translated into Nepali language. Finally, it was back translated into English with the guidance of two independent Nepalese HIV researchers. Questionnaire was then pretested among 15 HIV-positive individuals and assessed their received services at the ART clinic of Tribuvan University Teaching Hospital, Kathmandu, Nepal. Inconsistent questions were removed after pretesting. Additionally, sentence structure was also modified in the questionnaire to make it more suitable to the participants. After that, the final version was developed.

### Ethical Considerations

This study was approved by Research Ethics Committee of the University of Tokyo and the Ethical Review Board of Nepal Health Research Council, Nepal. National Center for AIDS and STD Control /Ministry of Health and Population also provided written permission to conduct this study. Written permission was also received from each study clinic.

Participation was voluntary. Before interview each HIV-positive individual was informed about the study objectives. Participants’ anonymity was maintained throughout the study. No personal identifiers were recorded. Furthermore, interview was conducted in confidential environment. At any stage, participants could decide not to participate in this study. Written informed consent was taken from each HIV-positive individual prior to conducting the interview.

### Statistical analyses and processing

Data was entered in EpiData version 3.1(EpiData Association Denmark). The entered data was then exported to STATA version 13.1(College Station, Texas, USA) for data analyses. The association was assessed between independent variables and HIV-positive individuals’ clinic attendance for pills pick-up by bivariate and multiple logistic regression analyses. Descriptive analysis was used to compare the characteristics of HIV-positive individuals. Chi-square and independent t-tests were used for descriptive analysis. Chi-square test and independent t-test were used for categorical and continuous variables, respectively.

In multiple logistic regression model, this study used the factors which were hypothesized to be associated with HIV-positive individuals’ clinic attendance for pills pick-up according to previous studies. These factors were age, sex, formal education, employment status, place of residence, commuting time, HIV status disclosure, ART regimen, clinical stage, tuberculosis diagnosis, perceived family support, participation in support programs, health status, and ART literacy [11, 12, 17, 18, 20, 25, 26, 36, 37].

Multicollinearity was not identified in multiple variable regression model. This conclusion was reached after calculating variance inflation factor. None of the variables had variance inflation factor greater than 10.0, the recommended value [48]. The statistical significance was set at p-value <0.05. In addition, goodness of fit test was satisfactory for the adjusted model (p = 0.549). We checked this by Hosmer- Lemeshow goodness of fit test.

## Results

### General characteristics of the participants

Table 1 shows the general characteristics of all HIV-positive individuals (n = 423). Their mean age was 39.7 years (SD 9.5) and 47.8% were men. Out of the total, 285 (67.4%) and 138 (32.6%) were categorized as inconsistent and regular clinic attendants, respectively. Commuting time to clinics was long amongst the inconsistent attendants. Those who spent more than 60 minutes commuting were significantly high among inconsistent attendants compared to regular attendants (p = 0.015). Family support score was significantly low among inconsistent attendants (p<0.001). Moreover, inconsistent attendees demonstrated a significantly low level of participation in support programs when compared to regular attendees (p = 0.031).

**Table 1:** General characteristics of the participants.

Characteristics	Overall	Regular attendants	Inconsistent attendants	p-value
		n (%)	n (%)	
**n (%)**	423	138 (32.6)	285 (67.4)	
**Age mean (SD)**	39.7 (9.5)	40.7 (10.2)	39.2 (9.1)	0.127
**Sex**				
Male	202 (47.8)	68 (49.3)	134 (47.0)	0.663
Female	221 (52.2)	70 (50.7)	151 (53.0)	
**Formal education**				
Yes	282 (66.7)	99 (71.7)	183 (64.2)	0.124
No	141 (33.3)	39 (28.3)	102 (35.8)	
**Employment status**				
Unemployed	154 (36.4)	52 (37.7)	102 (35.8)	0.705
Employed	269 (63.6)	86 (62.3)	183 (64.2)	
**Place of residence**				
Urban	277 (65.5)	98 (71.0)	179 (62.8)	0.096
Rural	146 (34.5)	40 (29.0)	106 (37.2)	
**Perceived family support**				
Low(0–22)	174 (41.2)	38 (27.5)	136 (47.7)	<0.001
Moderate(23–26)	105 (24.8)	23 (16.7)	82 (28.8)	
High(27–30)	144 (34.0)	77 (55.8)	67 (23.5)	
**Participating in support program**				
Yes	171 (40.4)	66 (47.8)	105 (36.8)	0.031
No	252 (59.6)	72 (52.2)	180 (63.2)	
**Commuting time**				
≤60 minutes	253 (59.8)	94 (68.1)	159 (55.8)	0.015
>60 minutes	170 (40.2)	44 (31.9)	126 (44.2)	

#### Health and HIV status related characteristics of the participants

Table 2 demonstrates the comparison of health and HIV status related characteristics between inconsistent and regular clinic attendants. Of the 423 participants, 4.9% self-rated their health status as excellent, then 24.4% very good, 40.7% good, and 30.0% fair. Among inconsistent attendants (n = 285), 44.2% self-rated their health status as good, compared to 33.3% among regular attendants (n = 138) (p = 0.001). ART related knowledge was significantly low among inconsistent attendants, such as the benefits of taking ART (p<0.001), limitations of ART (p<0.001), and the dangers of missing pills (p = 0.003).

**Table 2:** Health and HIV status related characteristics of the participants.

Characteristics	Overall	Regular attendants	Inconsistent attendants	p-value
	n (%)	n (%)	n (%)	
**HIV status disclosure (family or friends)**				
Yes	341 (80.6)	105 (76.1)	236 (82.8)	0.101
No	82 (19.4)	33 (23.9)	49 (17.2)	
**ART regimen**				
AZT^a^+3TC^b^+EFV^c^	58 (13.7)	20 (14.5)	38 (13.3)	0.842
AZT+3TC+NVP^d^	180 (42.6)	62 (44.9)	118 (41.4)	
TDF^e^+3TC+EFV	83 (19.6)	25 (18.1)	58 (20.4)	
TDF+3TC+NVP	102 (24.1)	31 (22.5)	71 (24.9)	
**Clinical stage**				
Stage I	72 (17.0)	17 (12.3)	55 (19.3)	0.349
Stage II	99 (23.4)	33 (24.0)	66 (23.2)	
Stage III	226 (53.4)	79 (57.2)	147 (51.6)	
Stage IV	26 (6.2)	9 (6.5)	17 (5.9)	
**Ever Tuberculosis diagnosis**				
Yes	87 (20.6)	24 (17.4)	63 (22.1)	0.261
No	336 (79.4)	114 (82.6)	222 (77.9)	
**Self-assessed health status**				
Excellent	21 (4.9)	14 (10.2)	7 (2.5)	0.001
Very good	103 (24.4)	30 (21.7)	73 (25.6)	
Good	172 (40.7)	46 (33.3)	126 (44.2)	
Fair	127 (30.0)	48 (34.8)	79 (27.7)	
**ART literacy**				
**Knowledge on the benefits of ART**				
Yes	309 (73.1)	122 (88.4)	187 (65.6)	<0.001
No	114 (26.9)	16 (11.6)	98 (34.4)	
**Knowledge on the limitations of ART**				
Yes	266 (62.9)	107 (77.5)	159 (55.8)	<0.001
No	157 (37.1)	31 (22.5)	126 (44.2)	
**Knowledge on the dangers of missing pills**				
Yes	311 (73.5)	114 (82.6)	197 (69.1)	0.003
No	112 (26.5)	24 (17.4)	88 (30.9)	

^a^ AZT- Zidovudine

^b^ 3TC-Lamivudine

^c^ EFV- Efavirenz

^d^ NVP- Nevirapine

^e^ TDF- Tenofovir.

#### Factors associated with regular clinic attendance for pills pick-up

Table 3 illustrates the results of bivariate and multiple logistic regression analyses. In an unadjusted model, HIV-positive individuals were more likely to attend the clinics regularly when they received high family support (score 27–30) (p<0.001). They were also more likely to attend them regularly when they had high ART literacy. Especially, knowledge on the benefits of ART (p<0.001), limitations of ART (p<0.001), and the dangers of missing pills (p = 0.004). They were also more likely to attend them regularly when they participated in support programs (p = 0.031). On the other hand, they were less likely to attend them regularly when they commuted more than 60 minutes to the clinics (p = 0.016) and when they self-rated their health status as being very good (p = 0.002), good (p = 0.001), and fair (p = 0.017).

**Table 3:** Factors associated with regular clinic attendance for pills pick-up among the people living with HIV in Nepal.

	Unadjusted model		Adjusted model	
Variables	OR	95% CI	AOR	95% CI
**Age**	1.01	0.99–1.03	1.01	0.98–1.04
**Sex**				
Male	1.00		1.00	
Female	0.91	0.60–1.37	1.12	0.64–1.98
**Formal education**				
Yes	1.00		1.00	
No	0.70	0.45–1.10	0.65	0.36–1.17
**Employment status**				
Unemployed	1.00		1.00	
Employed	0.92	0.60–1.40	0.96	0.55–1.67
**Place of residence**				
Urban	1.00		1.00	
Rural	0.68	0.44–1.06	1.06	0.58–1.95
**Commuting time**				
≤60 minutes	1.00		1.00	
>60 minutes	0.59	0.38–0.90*	0.53	0.30–0.93*
**HIV status disclosure(family or friends)**				
Yes	1.00		1.00	
No	1.51	0.92–2.48	1.14	0.62–2.10
**ART regimen**				
AZT+3TC+EFV	1.00		1.00	
AZT+3TC+NVP	0.99	0.53–1.86	1.34	0.63–2.84
TDF+3TC+EFV	0.81	0.40–1.67	0.87	0.37–2.02
TDF+3TC+NVP	0.82	0.41–1.64	0.93	0.41–2.11
**Clinical stage**				
Stage I	1.00		1.00	
Stage II	1.61	0.81–3.21	1.95	0.89–4.26
Stage III & IV	1.73	0.95–3.17	1.98	0.97–4.03
**Ever Tuberculosis diagnosis**				
Yes	1.00		1.00	
No	1.34	0.80–2.27	1.40	0.74–2.67
**Perceived family support**				
Low(0–22)	1.00		1.00	
Moderate(23–26)	1.00	0.55–1.80	0.84	0.44–1.60
High(27–30)	4.11	2.52–6.68***	3.98	2.29–6.92***
**Participating in support program**				
No	1.00		1.00	
Yes	1.57	1.04–2.37*	1.68	1.00–2.82*
**Self-assessed health status**				
Excellent	1.00		1.00	
Very good	0.20	0.07–0.55**	0.13	2. 0.04–0.44**
Good	0.18	0.06–0.48**	0.14	0.04–0.46 **
Fair	0.30	0.11–0.80*	0.21	0.06–0.70*
**ART literacy**				
**Knowledge on the benefits of ART**				
No	1.00		1.00	
Yes	3.99	2.24–7.10***	2.62	1.15–5.99*
**Knowledge on the limitations of ART**				
No	1.00		1.00	
Yes	2.73	1.72–4.34 ***	1.42	0.70–2.86
**Knowledge on the dangers of missing pills**				
No	1.00		1.00	
Yes	2.12	1.27–3.52**	1.47	0.78–2.79

*p<0.05

**p<0.01

***P<0.001.

In an adjusted model, HIV-positive people were more likely to attend the clinics regularly when they received high family support (Adjusted Odds Ratio [AOR] = 3.98, 95% CI = 2.29, 6.92) and when they participated in support programs (AOR = 1.68, 95% CI = 1.00, 2.82). They were also more likely to attend them regularly when they had knowledge on the benefits of ART (AOR = 2.62, 95% CI = 1.15, 5.99). On the other hand, they were less likely to attend them regularly when they commuted more than 60 minutes to the clinics (AOR = 0.53, 95% CI = 0.30, 0.93) and when they self-rated their health status as being very good (AOR = 0.13, 95% CI = 0.04, 0.44), good (AOR = 0.14, 95% CI = 0.04, 0.46), and fair (AOR = 0.21, 95% CI = 0.06, 0.70).

## Discussion

This is one of the first studies demonstrating the association between perceived family support of HIV-positive individuals and their clinic attendance for ARV pills pick-up. Of the 423 participants, only 32.6% attended the clinics regularly during the study period. They were more likely to attend them regularly when they received high family support, participated in support programs, and knew the benefits of ART. On the other hand, they were less likely to attend them regularly when they commuted more than 60 minutes from their community to the clinics and when their self-rated health status was better.

This study identified nearly 70% of inconsistent clinic attendances for pills pick-up, which is much higher than other settings, ranging from 6% to 40% [11, 12, 49, 50]. The high level of inconsistent attendance might be first explained by the low awareness about the definition of on-time pills pick-up among HIV-care providers. The definition was newly established in the new guidelines in 2014 [28]. This guideline allowed two days’ delay of the scheduled day for the pills pick-up, while previous one did not [27]. However, this study assessed clinic attendance on an assumption that the new 2014 guidelines were already passed through and implemented. Second, the high inconsistent rate could be attributed from the longer follow-up period of attendance (12 months) compared to other studies (3 to 7 months) [36, 38, 50]. Still, ongoing efforts should focus on reducing barriers to regular clinic attendance. Furthermore, effective strategies are required to make HIV-care providers more aware of on-time pills pick-up and updated ART guidelines at the operational level.

HIV-positive individuals were more likely to attend the clinics regularly when they received high family support. This result was consistent with the findings of other studies [39, 51]. However, a study in Ethiopia showed lower levels of ART medication adherence among those receiving family support [52]. Despite family support, people may miss their medication intake in working places and other places outside home. In contrast, family’s support might be more important during clinic visits. As clinic visit requires more time, travel expenses, and suitable environmental factors [53]. The physical support from a family is known to be helpful to ensure regular clinic attendance, it provides monetary support, transportation provision for commuting, and appointments reminding [17, 25, 26, 54–56]. Particularly in Nepal, a difficult geographic terrain may hinder smooth transportation. This support can often be indispensable for a HIV-positive people’s regular clinic attendance. Furthermore, a family’s psychological support is also important. Such support often provides HIV-positive people psychological strength and facilitate them to cope with their treatment fatigues. However, contrary to a family’s positive influence on regular clinic attendance, only 34% of participants reported such high family support. This could be attributed to existing socio-cultural context and rampant stigma and discrimination amongst them.

Furthermore, support program participants were more likely to attend the clinics regularly. This result was consistent with findings from studies in Tanzania and Cambodia [11, 56]. Support programs have encouraged them to attend clinics regularly in several settings [11, 56, 57]. Nepali ART guidelines have also strongly recommended support programs among them along with HIV care [27, 28]. The majority of clinics in Nepal has implemented programs, such as nutritional, psychosocial, and travel support. For example, distribution of wheat flour fortified with vitamins and minerals, provision of peer counseling in every clinic visit, and need-based partial or full reimbursement of travel expenses are provided under nutritional, psychosocial, and travel support, respectively. These programs were also being implemented at the selected ART clinics under this study.

People living with HIV who knew the benefits of ART were more likely to attend the clinics regularly. This result was consistent with previous studies [14, 58]. Increased knowledge of ART might have made them more aware of their role in HIV care. This empowers people living with HIV to highly engage themselves in their HIV treatment [13, 14, 58]. Knowledge of ART could assist HIV-positive people with various coping and self-efficacy skills [55].

High self-rated health status was negatively associated with regular clinic attendance. This result was consistent with a qualitative study in Africa [56]. People may skip appointments when they perceive themselves in good health because of the absence of HIV related symptoms. This could be due to their poor health literacy which has been also associated with self-assessed health status [59]. Poor health literacy may limit people living with HIV to understand their own health status, illness, and treatment outcomes. Moreover, their poor health literacy has also been associated with poorer medication adherence [60, 61] and ART discontinuation [58, 62].

In addition to increasing ART coverage, our results suggest that interventions should be implemented among HIV-positive individuals to improve their clinic attendance. These interventions should specifically focus on increasing health literacy, promoting family support, increasing support programs coverage, and ART literacy among them.

Our study has two major strengths. First, it examined the underexplored association between perceived family support and clinic attendance for ARV pills pick-up. Second, this is the first study in Nepal which measured clinic attendance for ARV pills pick-up among HIV-positive people. Findings of this study could be useful to national HIV response in similar Asian settings and elsewhere, too.

However, our results should be considered in the context of several limitations. First, the cross-sectional nature of our study limits casual inference. Second, information was not collected about CD4 cell count and viral load test results. Strong association has been demonstrated between regular clinic attendance and such treatment outcomes [49, 63]. In Nepal, however, it was difficult as only one center provides viral load testing and only 18 out of 61 ART clinics have CD4 testing services [24]. Service interruptions for CD4 test are common due to maintenance and logistic related problems. Because of these reasons, updated information was not collected. Third, this study did not include duration of HIV-positive people taking ART. In similar context, positive association has been demonstrated between duration on ART (more than 4 years) and clinic attendance among HIV-positive people [11]. To reduce its effect, participants were limited to those who started taking ART between 2012 and 2014. Fourth, this study did not measure HIV-care providers’ awareness of on-time pills pick-up. Although it directly influences HIV-positive individuals’ clinic visits for ARV pills pick-up, they may have been counted as missed attendance when they attended clinics a few days later from their scheduled day. Fifth, our study can only be generalized to similar socio-economic context. Finally, this study may be affected by social desirability bias; to reduce this, complete anonymity was assured to each participant.

## Conclusion

This study demonstrated low levels of regular clinic attendances for ARV pills pick-up among HIV-positive people in Nepal. They were more likely to attend them regularly when they received high family support, or were participating in support programs, or knew the benefits of ART. Whereas, they were less likely to attend them regularly when they self-rated their health status as better and had long commuting time to the ART clinics. These findings highlight the importance of incorporating family support, health literacy, support programs, and ART literacy with ongoing efforts to improve HIV care in Nepal. Particularly, health care providers should educate HIV-positive individuals about the importance of attending clinics regularly even in the absence of HIV-related symptoms. Moreover, there is an urgent need for the inclusion of family members in HIV care programs in Nepal.

## Supporting Information

S1 Appendix Survey Questionnaire. (DOCX)
