# Supplementary material for: Clinic Attendance for Antiretroviral Pills Pick-Up among HIV-Positive People in Nepal: Roles of Perceived Family Support and Associated Factors
Source: PLoS One. 2016 Jul 20;11(7):e0159382. doi: 10.1371/journal.pone.0159382 (PMC4954679; doi:10.1371/journal.pone.0159382)
Supplement: S1 Appendix — (DOCX) [file pone.0159382.s001.docx]

**Questionnaire (English Version)**

**Place of interview: …………………. Name of interviewer: ……………….**

**Date: …………**

**Starting time of interview………… Ending time of interview: ...……..**

| **Section 1: Socio-demographic characteristics** | | | |
| --- | --- | --- | --- |
| **No** | **Questions and filters** | **Coding categories** | **Skip** |
| **101** | How old are you?  (In completed years) |  |  |
| **102** | Gender | Male……………………………….1  Female……………………………..2  Other……………………………….3 |  |
| **103** | Have you ever attended school? | Yes………………...………….……1  No………………………………….2 | **If “NO” go 105** |
| **104** | If yes, what is the highest grade you completed? |  |  |
| **105** | Where do you presently live? | Urban………………...1……..……..  ( Specify municipality name)  Rural……..….2……………………...  ( Specify VDC name) |  |
| **106** | What is your current occupation? | Unemployed…………………..…..1  Agriculture………………………..2  Government employee……………3  Business…………………………..4  Student……………………………5  Others………………………(specify) |  |
| **107** | What is the total commuting time from your living place to ART center? | ……………….. (in mins, hours, days) |  |
| **108** | Are you participating in any HIV care and support programs? | Yes……………………..………….1  No…………………………..……..2  Don’t know……………………….98 |  |
| **109** | If yes, could you specify the program you are participating in? | Community and home based care programs…………………………..1  Community care centers….……….2  HIV-positive led community lead care and support programs…………….3  NGO……………………………….4  Others……………………….(specify) |  |
| **Information related to earthquakes** | | |  |
| **110** | Were you or your family member(s) affected by the earthquake(s) on 25^th^ April and 12^th^ May, 2015? | Yes…………………………………1  No………………………………….2 | **If “NO” go to 112** |
| **111** | If yes, could you please tell us what happened to you or your family member(s)? | ………………………………...…… |  |
| **112** | Were any ART services affected by the recent earthquake(s)? | Yes…………………………….…..1  No…………………………………2 | **If “NO” go to 201** |
| **113** | If yes, how were the ART services affected? | Interruption in antivirals supply…1  Damage in ART center…………..2  Lack of attendance of health care provider…………………………..3  Others…………………….(specify) |  |

| **Section 2: Clinical characteristics** | | | |
| --- | --- | --- | --- |
| **No** | **Questions and filters** | **Coding categories** | **Skip** |
| **201** | Have you ever been diagnosed with TB? | Yes………………………1  No……………………….2  Don’t know……. ………99 |  |
| **202** | If yes, what type of TB did you suffer from? | Pulmonary………………1  Extra-pulmonary………..2  …………………..  (Specify TB type) |  |
| **203** | Have you disclosed your HIV status with someone other than HIV care providers ? (family or friends) | Yes………………………1  No……………………….2 |  |
| **204** | If Yes, with whom you have disclosed your HIV status?( Multiple answers possible) | Partner or Spouse……..1  Family members….......2  Others…………..(specify) |  |

| **Section 3: Self-assessed health status of the participants** | | | |
| --- | --- | --- | --- |
| **NO** | **Questions and filters** | **Coding categories** | **Remarks** |
| **301** | In general, would you say your health is: | Excellent………………..1  Very good………………2  Good……………………3  Fair……………………..4  Poor…………………….5 |  |

| **Section 4: Perceived family support**  For each item, please ask participants to rate how true each statement is for their own family on a four-point Likert scale ranging from “Not at all” (0), sometimes(1), often (2), to “All the time” (3). | | | |
| --- | --- | --- | --- |
| **NO** | **Questions and filters** | **Coding categories** | **Skip** |
| **401** | In past twelve months,  How much does your family show love and caring for you? | Not at all………0  Sometimes…….1 Often………… 2  All the time……3 |  |
| **402** | In past twelve months, How much do you feel disliked by your family? | Not at all………0  Sometimes…….1 Often………… 2  All the time……3 |  |
| **403** | In past twelve months, How much do you feel you have an important role in your family? | Not at all………0  Sometimes…….1 Often………… 2  All the time……3 |  |
| **404** | In past twelve months, How much do you feel (emotionally) distant from your family? | Not at all………0  Sometimes…….1 Often………… 2  All the time……3 |  |
| **405** | In past twelve months, How much are you involved in family decision making? | Not at all………0  Sometimes…….1 Often………… 2  All the time……3 |  |
| **406** | In past twelve months, How much are your basic needs (food and clothes) met in your family? | Not at all………0  Sometimes…….1 Often………… 2  All the time……3 |  |
| **407** | In past twelve months, How much support do you get from family when you are sick? | Not at all………0  Sometimes…….1 Often………… 2  All the time……3 |  |
| **408** | In past twelve months, How much has your family physically (beaten) hurt you? | Not at all………0  Sometimes…….1 Often………… 2  All the time……3 |  |
| **409** | In past twelve months, How much can you share your feelings with your family? | Not at all………0  Sometimes…….1 Often………… 2  All the time……3 |  |
| **410** | In past twelve months, How much does your family exploit you (such as for labor in the house and/or farming)? | Not at all………0  Sometimes…….1 Often………… 2  All the time……3 |  |

| **Section 5: Treatment literacy** | | | |
| --- | --- | --- | --- |
| **NO** | **Questions and filters** | **Coding categories** | **Skip** |
| **501** | What are the benefits of taking ART?   1. Reduces viral load 2. Treats infections 3. Increase CD4 count 4. Prevent mother to child transmission | Correct……..1  Incorrect……2  Don’t know…3 |  |
| **502** | What are the limitations of taking ART?  (Probe: what ART cannot do?)   1. It cannot cure HIV 2. Should be taken life long 3. Patients on ART can still transmit HIV 4. Has short and long term side effects | Correct……..1  Incorrect……2  Don’t know…3 |  |
| **503** | Could you tell us the dangers of forgetting to take medicine?   1. Low CD4 count 2. Increase in number of infections 3. Disease becomes severe | Correct……..1  Incorrect……2  Don’t know…3 |  |

**From treatment and care files of the participants**

| **1. Clinic attendance**  July, 2014 to June, 2015  Use treatment and care register to assess the following information. | | | |
| --- | --- | --- | --- |
| **NO** | **Scheduled day (Day/Month/Year)** | **Outcome of scheduled day**  **“Adhered” or “Missed”** | **Remarks** |
|  |  |  |  |
|  |  |  |  |
|  |  |  |  |
|  |  |  |  |
|  |  |  |  |
|  |  |  |  |
|  |  |  |  |
|  |  |  |  |
|  |  |  |  |
|  |  |  |  |
|  |  |  |  |
|  |  |  |  |

| **2. Missed days**  July, 2014 to June, 2015  Use treatment and care register to assess the following information. | | | |
| --- | --- | --- | --- |
| **SN** | **Missed visit ( “YES” or “NO”)** | **Date of missed day** | **Remarks** |
|  |  |  |  |
|  |  |  |  |
|  |  |  |  |
|  |  |  |  |
|  |  |  |  |
|  |  |  |  |
|  |  |  |  |
|  |  |  |  |

1. Date of ART start………………………………………
2. Last date of follow up: ……………………………
3. Client status

- Missing Yes…………….1 No…………..2
- If yes, date of missing DD/MM/ YY ..……………………
- Active on ART Yes…………….1 No…………..2

1. WHO clinical staging: WHO stage I …….……..1

WHO stage II .…………..2

WHO stage III ………..….3

WHO stage IV …………..4

1. ART regimen currently taking: …………………………….
